# Growth of exclusively breastfed small for gestational age term infants in the first six months of life: a prospective cohort study

**DOI:** 10.1186/s12887-021-03080-6

**Published:** 2022-02-01

**Authors:** Neti Nurani, Tunjung Wibowo, Rina Susilowati, Janatin Hastuti, Madarina Julia, Mirjam M. Van Weissenbruch

**Affiliations:** 1 Department of Child Health, Faculty of Medicine, Public Health and Nursing, Universitas Gadjah Mada/ Dr. Sardjito General Hospital, Yogyakarta, Indonesia; 2 Department of Histology and Cell Biology, Faculty of Medicine, Public Health and Nursing, Universitas Gadjah Mada, Yogyakarta, Indonesia; 3 Department of Health Nutrition, Laboratory of Bioanthropology & Paleoanthropology, Faculty of Medicine, Public Health, and Nursing, Universitas Gadjah Mada, Yogyakarta, Indonesia; 4 Department of Neonatology, Emma Children’s Hospital, Amsterdam UMC, Vrije Universiteit Amsterdam, De Boelelaan 1117, Amsterdam, 1081 HV The Netherlands

**Keywords:** Growth, Exclusively breastfed, Small for gestational age, Infant

## Abstract

**Background:**

Compared to their appropriate-for-gestational-age (AGA) peers, small-for-gestational-age (SGA) infants are prone to growth deficits. As the first 6 months of exclusive breastfeeding is generally recommended, it is essential to understand how this intervention might impact SGA infants’ growth. This study aims to assess growth of exclusively breastfed SGA term infants in the first 6 months of life.

**Methods:**

A prospective cohort study was conducted on term infants born in Dr. Sardjito General Hospital and two private hospitals in Yogyakarta, Indonesia. SGA was defined as birth weight less than the 10th percentile according to Fenton criteria. Weight, length, and head circumference (HC) were measured at birth and monthly until 6 months old.

**Results:**

A total of 39 AGA and 17 SGA term infants who were exclusively breastfed in their first 6 months were included and followed. In SGA compared to AGA, birth weight, length, and HC (mean ± SD) were significantly lower (*p* < 0.001). During the first 6 months, the SGAs grew in weight and length in parallel with the AGAs. At sixth months of age, the weight and length (mean ± SD) of the SGAs were significantly lower compared to the AGAs (*p* < 0.001). However, HC (mean ± SD) of SGAs grew significantly faster than the AGAs (*p* < 0.005). At sixth months of age, there were no significant differences in HC between the two groups (*p* = 0.824).

**Conclusions:**

In the first 6 months, exclusively breastfed SGA term infants, in contrast to weight and length, only show catch up growth in HC, leading to HC comparable to their AGA peers at the age of 6 months.

**Supplementary Information:**

The online version contains supplementary material available at 10.1186/s12887-021-03080-6.

## Background

Small-for-gestational-age (SGA), defined as infants whose birth weight were less than 10th percentile of the reference, was widely known to have higher risk for perinatal morbidity, growth restriction leading to persistent short stature, neurodevelopmental problems such as low intelligence quotient (IQ), and pubertal disorder in later life [1–4]. Recent studies also observed that SGA infants have a higher risk for metabolic disorders later in life, with metabolic syndrome prevalence during childhood and adolescent doubled in SGA subjects than in AGA subjects (OR 4.08, 95% CI 1.48 to 11.22) [5–7].

In 2012, the prevalence of infants born SGA was estimated to be 23.3 million in low and middle-income countries [8]. South or South East Asia had the most SGA births in the world (62.7%), compared to Australia with only 9.4% [8, 9]. In South East Asia, SGA infants born with and without low birth weight were approximately 20% of total live births [8].

Catch up growth, defined as body weight reaching the 10th percentile of the corrected age for SGA children, in SGA infant was regarded as advantageous in maximizing neurodevelopment, enhancing immune function, and achieving final adult height [10, 11]. However, it also has certain disadvantages. Excessive rapid growth of SGA infants had been related to increased risk of metabolic syndrome and cardiovascular disorders later in life [1, 6, 12]. Catch up growth (CUG) of SGA infants usually takes place in 6 months- to 2 years of age [13–16]. Studies in Asia, however, show earlier CUG. A study in the Philippines revealed limited higher rates of weight-for-length gain in the first month of life, followed by little catch up growth thereafter [17]. In addition, children born full-term SGA in China display rapid growth after birth until 6 months of age [18].

Maternal characteristics such as age, parity status, educational level, and medical condition (e.g., nutritional status, anemia, illness during pregnancy) have been variously shown to influence intrauterine and postnatal growth [19]. Variation in feedings practice had also been shown to influence growth. Compared to their formula fed peers, exclusively breastfed infants had been shown to grew better [20].

Exclusive breastfeeding, defined as giving infants only breast-milk without other food or water for the first 6 months age, is the cornerstone of child growth and development as it provides essential and optimal nutrition for child [21]. However, its practice in mothers of SGA may face more challenges than in AGA mothers [20]. Concerns regarding the infants’ size often cause parents or health workers to add formula milk to the infants’ diet.

This study aims to evaluate growth of exclusively breastfed SGA term infants in the first 6 months compared to appropriate-for-gestational-age (AGA) term infants. We hypothesized that the exclusively breastfed SGA grew better than their AGA peers to catch up for their intra-uterine growth deficits.

## Methods

### Study design

This is a prospective longitudinal observational cohort study of exclusive breastfeeding in relation to growth in term newborns from birth until the age of 6 months.

### Subjects

Term infants who were born in the Perinatology ward/Neonatal Intensive Care Unit (NICU) of Sardjito General Hospital and two private hospitals (Bhakti Ibu and Sadewa Maternal hospital) in Yogyakarta were included in this study. Yogyakarta was an urban city located in a province in the middle of Java Island, Indonesia. Based on Indonesia Demographic and Health Survey 2017, the crude birth rate in Yogyakarta was 15.3 [22].

Ninety-one infants were enrolled consecutively between July 2018 to October 2019. Infants were eligible for study participation if they were born with a gestational age of ≥37 until 41 6/7 completed weeks and exclusively breastfed [23]. The exclusion criteria were congenital anomalies, conditions preventing mothers to nurse their babies, such as human immunodeficiency virus (HIV)/acquired immunodeficiency syndrome (AIDS)-infection, mastitis in both breasts, severely ill mothers (sepsis, decreased consciousness, admission to the intensive care unit, etc.), and mothers with acute depression or other psychiatric disorders.

For all eligible infants, written informed consent was obtained from both parents before enrollment. Ethical approval was obtained from the Medical and Health Research Ethics Committee of Faculty of Medicine, Public Health and Nursing, Universitas Gadjah Mada.

### Data collection

#### Maternal characteristics

Maternal characteristics consisted of maternal age, parity, delivery mode, education, occupation, and history of cigarette exposure, were obtained using questionnaires collected after delivery. Mothers self-reported cigarette exposure data from whether she smoked during pregnancy (active smoker) or her family smoked inside the house (passive smoker). Passive smoker was a term used to refer to someone who breathes other people’s smoke [24]. A former study carried by Liu et al., defines passive smokers based on their affirmative response to questions whether there were smokers living in participants’ families, at the workplace, or during adulthood [25].

Pre-pregnancy body mass index (BMI) was calculated based on weight of mother before pregnancy divided by square of height measured at antenatal care visit or after delivery (in kg/m2). Assessment of gestational age was based on ultrasound examination in the first trimester and/or dubowitz score in case there was no ultrasound examination. Hemoglobin level of the mother was measured just before delivery.

#### Infant characteristics

Weight, length, and head circumference (HC) of the infants were measured by trained research assistants within 24 h after birth and then every month (within 1 week after completion of the month) until the age of 6 months. The trained research assistants were tested for interrater reliability to avoid measurement bias. Infant-mother pairs went to hospital or home visited for follow up study.

Infants were weighed naked on a digital weighing scale (Seca 272) to the nearest 1 g. Infantometer (Seca 210) was used to measure the infants’ length to the nearest 1 mm. Head circumference was measured to the nearest millimeter using a non-stretch plastic measuring tape encircling the head at the occipital protuberance level posteriorly and supraorbital ridges anteriorly.

Intrauterine growth status was classified using PediTools Fenton 2013 [26]. Infants were considered SGA if their birth weight was less than the 10th percentile of the reference. They were classified as AGA if their birth weight was at or above the 10th percentile. The infants were classified as symmetrical SGA when all of the birth parameters (weight, length, and head circumference) were less than the 10th percentile of the reference and asymmetrical SGA if only body weight was less than the 10th percentile of the reference [27].

### Infants’ feeding record

Information on the infant’s feeding was documented at monthly visits. We used the 24-h recall to determine breastfeeding status as suggested by the WHO [28]. However, in addition to that, as we were also interested in assessing whether the infants received anything besides breastmilk during the month of the follow-up visit, we had also inquired about any food or drink that might be given on other days during the month. We excluded infants who received anything other than breastmilk [28].

### Statistical analyses

Continuous variables were presented as mean ± standard deviation (SD), while categorical variables were presented as numbers (%). Continuous variables were tested for skewedness and kurtosis. Normally distributed groups were compared using independent t-test. Categorical variables were compared using Chi-square test.

We used linear regression analyses to assess predictors of the infants’ weight, length, and head circumference at 6 months as the outcome variables. We did the analyses in two steps: (1) univariate linear regression analyses with weight, length and head circumference at birth as the predictors and sex, maternal height, maternal age, maternal pre-pregnancy BMI, cigarette exposure, etc. as the potential confounders and (2) the construction of multivariate models from independent variables with *p*-value of ≤0.25 in the univariate analyses [29–31] SPSS program version 22 was used to analyze the data. Statistically significant was defined when *p* < 0.05.

## Results

**Fig. 1:**
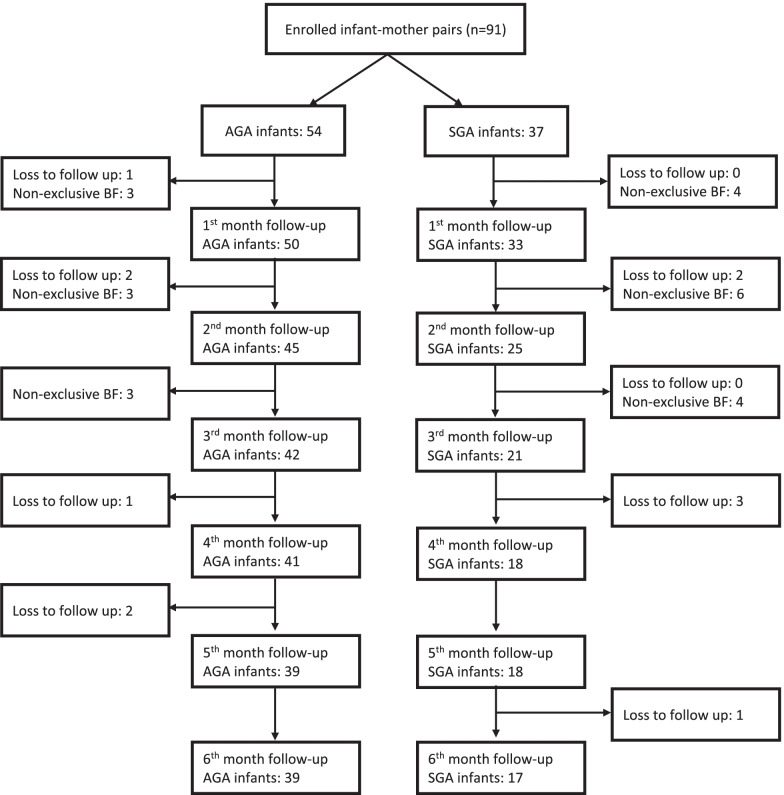
Flow chart of study participants

In the primary analysis, 91 term infants were included. However, only 56 (58.9% male) consisted of 39 AGA and 17 SGA infants completed the 6 months follow-up. Six (11.1%) of the AGA and 6 (16.2%) of the SGA were lost to follow up because they did not attend the follow-up session due to move from their former home or could not be reached by their phone number. Nine (16.7%) of the AGA and 14 (37.8%) of the SGA had to be excluded from the study because they were no longer exclusively breastfed. The flow diagram showing the exclusion of subjects during the study was presented in Fig. 1. Supplementary Table 1 showed no significant differences in the baseline characteristics of infants included in the final analyses vs. those lost to follow up or excluded due to failure of exclusive breastfeeding. However, the table also showed that those who were excluded were more likely to born with c-section, albeit not statistically significant.

SGA infants had significantly lower mean of weight, length, and head circumference at birth than AGA (2244.5 ± 387.4 g vs. 3143.4 ± 339.8 g, 45.4 ± 2.9 cm vs. 49.1 ± 1.9 cm, 31.5 ± 1.5 cm vs 33.96 ± 1.4 cm, respectively). There was no significant difference in gestational age between the AGA and the SGA. Fenton standard deviation scores of birth weight, length, and head circumference of SGA group were significantly lower compared to the AGA group (*p* = < 0.001). SGA mothers were younger, had lower pre-pregnancy BMI, and had lower height than AGA groups. Most of the mothers in this study were multiparous women. Half of the subjects were born through caesarean section. Mother’s age, parity, education, occupation, pre-pregnancy BMI, height, cigarette exposure, and anemia were not significantly different among the two groups (Table 1). Cigarette exposures were mostly as passive smokers. There were no significant differences in the number of cesarean sections and spontaneous deliveries between the two groups.

**Table 1:** Characteristics of the study population

Variable	AGA *n* = 39	SGA *n* = 17	*P*-value^a^
**Infant characteristics at birth**			
Sex (n, %)			
Male	21 (54)	12 (71)	0.37
Female	18 (46)	5 (29)	
Weight (g, mean ± SD)	3143.4 ± 339.8	2244.5 ± 387.4	< 0.001
Fenton SDS of weight (mean ± SD)	−0.2 ± 0.6	−2.1 ± 0.7	< 0.001
Length (cm, mean ± SD)	49.1 ± 1.9	45.4 ± 2.9	< 0.001
Fenton SDS of length (mean ± SD)	− 0.2 ± 0.9	− 1.5 ± 1	< 0.001
Head circumference (cm, mean ± SD)	33.96 ± 1.4	31.5 ± 1.5	< 0.001
Fenton SDS of head circumference (mean ± SD)	− 0.8 ± 0.9	− 1 ± 1.3	< 0.001
Gestational age (week, mean ± SD)	38.74 ± 0.9	38.5 ± 1.2	0.48
**Maternal characteristics**			
Age (year, mean ± SD)	32 ± 4.8	30 ± 5.7	0.15
Parity (n, %)			
Primi	12 (31)	8 (47)	0.36
Multipara (≥2)	27 (69)	9 (53)	
Mode of delivery (n, %)			
Caesarean Section	18 (46)	9 (53)	0.77
Spontaneous	21 (54)	8 (47)	
Education (n, %)			
Low (≤ 12 years)	13 (33)	7 (41)	0.76
High (> 12 years)	26 (67)	10 (59)	
Occupation (n, %)			
Employee	26 (67)	10 (59)	0.76
House wife	13 (33)	7 (41)	
Pre-pregnancy BMI (kg/m^2^, mean ± SD)	23.05 ± 3.3	22.54 ± 4.1	0.66
Height (m, mean ± SD)	1.57 ± 0.0	1.54 ± 0.0	0.09
Cigarette exposure (n, %)			
Yes	11 (28)	2 (12)	0.30
No	28 (72)	15 (88)	
Anemia (n, %)			
Yes (Hb < 11 g/dL)	15 (38.5)	6 (35.4)	0.53
No (Hb ≥11 g/dL)	24 (61.5)	11 (64.7)	

^a^Independent t test was used for continuous variables, which are presented as mean ± SD. Categorical variables were compared using Chi-square test, which are presented as numbers (%)

**Fig. 2:**
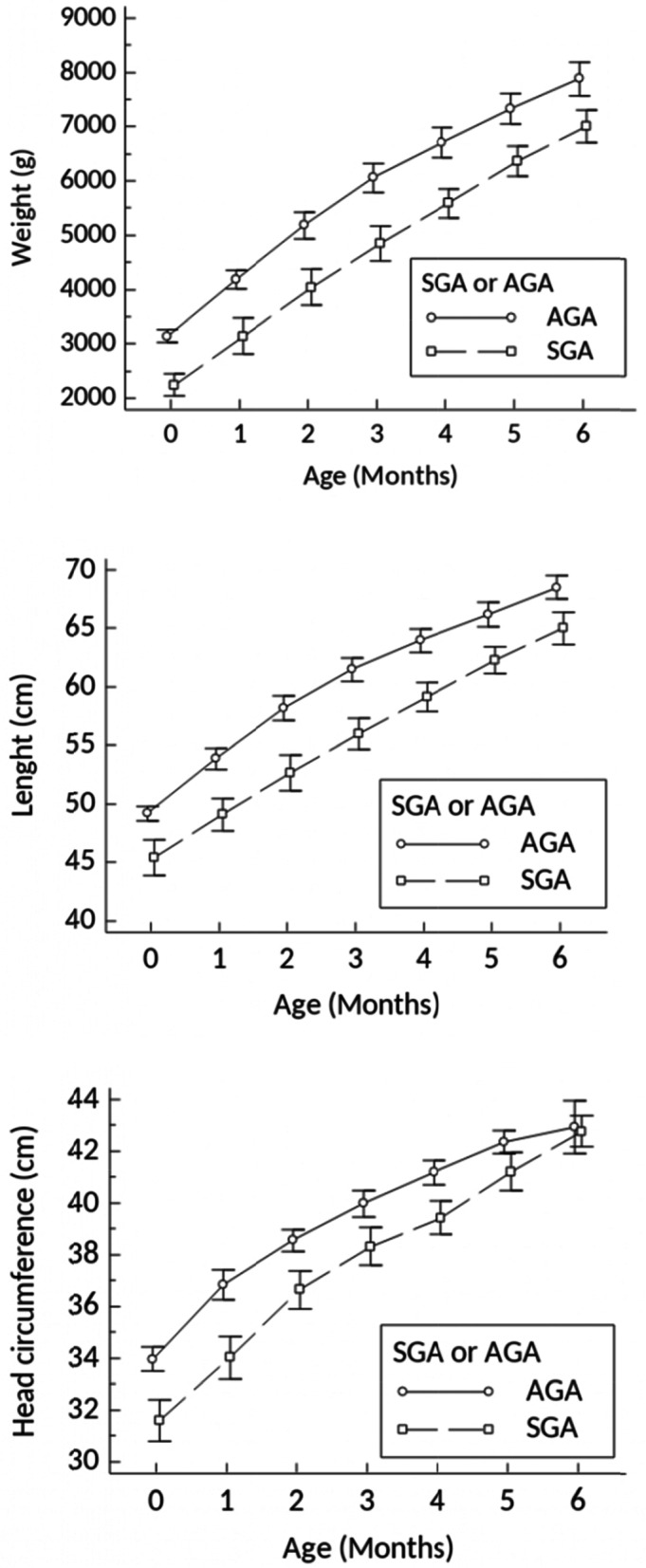
Changes of weight, length, and head circumference from birth until the age of 6 months

Growth in weight, length, and head circumference in the first 6 months between the two groups are shown in Fig. 2. The SGAs grew in parallel with the AGAs. At the age of 6 months, the mean of weight and length of the SGAs were still significantly lower than the AGAs (7009.4 ± 593.4 g vs 7880.5 ± 938.8 g and 65 ± 2.7 cm vs 68.5 ± 3.1 cm, respectively). Interestingly, there was no significant difference in the mean head circumference at the age of 6 months between the two groups, 42.8 ± 1.2 cm in SGAs vs. 42.9 ± 3.2 cm in AGAs. This indicates that the head circumference of the SGAs grew faster than the AGAs as the head circumference of SGAs at birth was significantly smaller than AGAs (Table 2).

**Table 2:** Comparison of weight, length, and HC at birth and at 6 months

Characteristics	Time	Term SGA (mean ± SD)	Term AGA (mean ± SD)	***P***-value^**a**^
**Weight (g)**	Birth	2244.5 ± 387.4	3143.4 ± 339.8	**< 0.001**
	6 months	7009.4 ± 593.4	7880.5 ± 938.8	**< 0.001**
**Length (cm)**	Birth	45.4 ± 2.9	49.1 ± 1.8	**< 0.001**
	6 months	65 ± 2.7	68.5 ± 3.1	**< 0.001**
**Head circumference (cm)**	Birth	31.5 ± 1.5	33.9 ± 1.4	**< 0.001**
	6 months	42.7 ± 1.1	42.9 ± 3.1	0.824

^a^Data were analysed using independent t test

Linear regression analysis, as seen in Table 3, showed that the infants’ gender, intrauterine growth status, and weight at birth were associated with weight at 6 months, while their length at 6 months was associated with the infant’s gender, intrauterine growth status, length at birth, and maternal height. Factors associated with infant’s HC at 6 months include the infant’s gender and HC at birth.

**Table 3:** Linear regression analyses for factors associated with the infants’ weigh, length and head circumference at 6 months. Multivariate model A using intrauterine growth status as predictor, while multivariate model B using birth weight, length or HC as predictor, respectively

Variables	Univariate			Multivariate Model A			Multivariate Model B		
	Unstandardized β	95%CI	*P*-value	Unstandardized β	95%CI	*P*-value	Unstandardized β	95%CI	*P*-value
**Weight at 6 months**									
Sex (1 = male, 0 = female)	646.3	163.4-1129.1	0.010	714.9	276.5-1153.4	0.002	682.5	307.7-1057.3	0.001
Intrauterine Growth (1 = SGA; 0 = AGA)	− 871.1	− 1367.1-(− 375.1)	0.001	− 886.9	− 1352.1-(− 421.8)	< 0.001			
Weight at birth	1.10	0.7-1.5	< 0.001				1.1	0.7-1.5	< 0.001
Gestational age	223.7	−18.5-465.9	0.070	38.6	− 176.8-253.9	0.721	− 146.3	− 346.9-54.3	0.149
Maternal height	6061.3	1644.3-10,478.3	0.008	3275.8	− 751-7302.6	0.109	1777.2	− 1791.4-5345.9	0.322
Maternal age	25.3	−24.2-74.7	0.310						
Maternal pre-pregnancy BMI	20.5	−49.7-90.7	0.561						
Adjusted *R*^2^					0.350			0.511	
**Length at 6 months**									
Sex (1 = male, 0 = female)	1.8	0.1-3.6	0.044	2.2	0.6-3.8	0.007	1.8	0.4-3.3	0.012
Intrauterine Growth (1 = SGA; 0 = AGA)	−3.5	−5.2-(−1.7)	< 0.001	−3.3	−5-(−1.6)	< 0.001			
Length at birth	0.7	0.4-0.9	< 0.001				0.6	0.4-0.9	< 0.001
Gestational age	0.6	−0.3-1.4	0.191	−.1	− 0.9-0.6	0.755	− 0.5	− 1.2-0.2	0.191
Maternal height	26.8	11.4-42.2	0.001	17.7	3.1-32.4	0.018	17.3	3.6-31.1	0.014
Maternal age	0.1	−0.1-0.3	0.235	.02	−0.1-0.2	0.824	0.05	−0.1-0.2	0.464
Maternal pre-pregnancy BMI	−0.03	− 0.3-0.2	0.759						
Adjusted *R*^2^					0.368			0.437	
**Head Circumference at 6 months**									
Sex (1 = male, 0 = female)	0.9	0.3-1.7	0.005	0.9	0.3-1.7	0.007	0.8	0.1-1.4	0.019
Intrauterine Growth (1 = SGA; 0 = AGA)	−0.7	−1.5-0.1	0.075	−0.8	−1.5-(− 0.1)	0.022			
Head circumference at birth	0.3	0.2-0.5	< 0.001				0.3	0.1-0.5	0.002
Gestational age	0.3	−0.01-07	0.056	0.1	−0.2-0.5	0.348	−0.05	− 0.4-0.3	0.758
Maternal height	3.8	−2.9-10.4	0.261						
Maternal age	−0.01	−0.1 − 0.1	0.78						
Maternal pre-pregnancy BMI	-0.1	−0.2-0.04	0.22	−0.05	−0.1-0.04	0.244	−0.05	− 0.1-0.03	0.231
Adjusted *R*^2^					0.200			0.266	

## Discussion

Our study showed that during the first 6 months of life breastfed full-term SGA infants grew in weight and length in parallel to their AGA peers, resulting in the persistence of a smaller size at the age of 6 months. On the other hand, their head circumference grew faster than the term AGAs, resulting in a similar head circumference at the age of 6 months [32].

Lucas et al. in 1997 observed that breastfed term SGA infants grew faster in weight, length, and head circumference than formula-fed term SGA infants [32]. However, he did not compare these results with the growth of term AGA infants [32]. A study in China showed that term SGA infants catch up in weight and head circumference by the age of 12 months, but not in length. In this study, information on infant feeding was not reported [18]. De Zegher et al. in 2013 reported that at 12 months of age, breastfed SGA term infants are still smaller in weight and length compared to their breastfed AGA term peers, however in that study the growth of head circumference was not mentioned [33].

Head circumference is commonly used as an indicator of brain size in infancy and early childhood. Postmortem studies of infants and a Computed Tomography (CT) imaging study of neonates with medical complications have found significant positive correlations between head circumference and brain size [34, 35]. A study in United States of America demonstrated a strong correlation between head circumference and brain volume in children at the age of 6 years and younger [36].

The human brain’s growth velocity is higher in the postnatal period than in the intrauterine period, particularly during the first 6 months of life [14]. Gale et al. reported that brain growth during early childhood is more important than intrauterine growth in determining cognitive function at 9 years of age [37]. SGA infants have a higher rate of head growth velocity than AGA infants in the first half of infancy as compensation to nutritional insult during fetal life, in order to catch up postnatally compared to their normal peers [38]. In line with the latter, our observation showing a more rapid head growth of breastfed SGA infants compared to their term AGA peers is important in view of the recognized association between growth retardation and later neurodevelopmental abnormalities [39].

The rapid head growth observed in our study might be explained by the fact that all of the infants were asymmetric SGAs. Kaur et al. found that the increase in head circumference was different among asymmetric and symmetric full-term SGA infants. Better growth in head circumference is attained in asymmetric SGA infants than in symmetric SGA counterparts during the first postnatal year of life; this finding may be attributed to the continuation of ‘brain sparing’ experienced by asymmetric SGA babies during prenatal life [38].

The catch up in growth of term SGA infants varied between studies. Some reported catch up growth as early as 3 to 6 months, while others failed to observe catch up growth until the end of the first year [17, 18, 40]. In our study, until the end of the sixth month, there were no signs of catch up in weight and length because the SGAs seemed practically grow in parallel with their AGA peers. Further follow-up is certainly needed.

Compared to symmetrical intrauterine growth restriction (IUGR), asymmetrical IUGR has a better prognosis for growth because their cell number is normal. Their only defect is in the cell size [41]. McIntosh and Stenson [42] also remarked that infants with asymmetric growth retardation usually catch-up to within the normal centiles after birth. In contrast, infants with symmetrical SGA or proportionate growth retardation, which have fewer cells at birth, will remain small later in life [39].

Several possible mechanisms were considered which might result in the better catch-up growth seen in breastfed SGA infants, including higher food intake, specific nutrients in breast milk, better nutrient absorption, non-nutrient factors in breast milk, or a combination of these factors [32]. The most popular model of catch up growth is the neuroendocrine hypothesis. The hypothesis explained that catch up growth resulting from conflicting action of growth hormone (GH) and ghrelin. Oxygenic ‘hunger signal’ of ghrelin increase the GH level. Ghrelin level increase in intrauterine and postnatal malnutrition which is associated with increased rates of catch up growth. Poor intrauterine growth (i.e. SGA) leads to an increase of the ghrelin level at birth. Persistent high level of ghrelin at 3 months of age, which occurred in SGA infants, is associated with greater catch-up growth [43].

Catch up growth in SGA infants is also influenced by growth hormone/insulin-like growth factor (GH/IGF-1) that has a major role in fetal growth as well as in infant and child growth [10]. An increase in GH is usually followed by an increase of IGF-1 level [43]. Abnormalities in GH/IGF-1 axis have been reported in SGA children as SGA infants have one SD lower mean serum level IGF-1 and IGF-binding protein-3 than AGA infants at birth. However, when they show catch-up growth, the SGA children have higher IGF-1 level than the AGA children. It was postulated that higher basal GH levels are a factor of early catch up growth. SGA children with inadequate postnatal catch up growth will remain short throughout childhood and have reduced adult height [10].

Exclusive breastfeeding is recommended for full-term children, regardless of their weight [44]. Previous studies reported that breastfeed SGA infants grew better compared to their formula-fed peers [32, 40]. Zegher et al. found that there was no significant difference in weight, length, lean mass, and fat mass between breastfed SGA (SGA-BRF) and formula-fed SGA (SGA-FOF) at 4 months of age [33, 44]. However, their study showed that SGA-FOF at 4 months old had higher IGF-1 levels than SGA-BRF, followed by higher fat mass without differences in length and lean mass [33]. This finding suggests that IGF-1 can stimulate adipose tissue expansion, not only for somatotropic growth, and IGF-1 seems to be sensitive to nutritional influences [33, 45]. As SGA-FOF have higher IGF-1 level, they may have a higher risk for cardiovascular and metabolic disease in later life, making breastfeeding the optimal choice of feeding for all infants [45, 46].

This study observed a high percentage of caesarian section (c-section), a condition that may partly explain the high rate of drop out in exclusive breastfeeding. The rate of c-section in the city of Yogyakarta in 2018 was 23,1%. The average rate of c-section in Indonesia was higher among urban women (22.1%) compared to rural women (12.4%) [47]. Among South East Asian countries participating in the South East Asia-Optimizing Reproductive and Child Health in Developing countries (SEA-ORCHID) project, Indonesia’s c-section rate was the second highest [48].

In this study, infant’s gender regardless of being born SGA or AGA is significantly and independently associated with weight, length, and HC at 6 months. Male infants, irrespective of their birth weight, length, and HC, are heavier, longer, and have bigger HC at 6 months than those of female infants. A similar finding was observed from a study in China. Male infants grow faster than females, up to the age of 12 months [18].

A study in the Philippines showed that the growth standard deviation scores (SDS) of male SGA infants are worse than those of female SGA infants when converting weight, length, or HC are to SD Scores of certain growth references. It was supposed that compared to their peers, the growth of male SGA infants might be more seriously affected [17].

Although Fig. 2 previous analyses showed that at 6 months of age, the HC of the SGA did not differ significantly from those of the AGA, the multivariable analyses showed that individual HC at birth was still influencing the HC at 6 months of age. It seems that, albeit HC of the SGA has grown significantly faster than those of the AGA, the smaller HC were still smaller at 6 months [18].

This study also showed that in contrast to weight, length at 6 months was independently associated with maternal height. This association is not easy to explain since maternal height might also be related to maternal education or socio-economic status, apart from its possible association with genetic factor [17, 49].

To the best of our knowledge, this is the first observational study describing the role of exclusive breastfeeding in growth of HC in term SGA infants of Javanese origin, Indonesia’s biggest ethnicity. Moreover, the results of this study have to be considered as recommendation for exclusive breastfeeding in term SGA infants, especially in Indonesia as one of the developing countries.

However, we do realize that the small sample size and the high number of both lost to follow up and excluded participants were the limitation of our study. It was not easy to convince mothers to exclusively breastfed for a full period of 6 months, especially in the SGA group. Similar situations were also observed in previous studies [50]. Comparison of the characteristics between infants who completed the 6 months follow up vs. who were dropped out did not show any significance difference.

Further follow up is certainly needed because by the age of 6 months, there was not yet any sign of catch up growth in length and weight.

## Conclusions

In conclusion, in the first 6 months of life, exclusively breastfed SGA term infants only show catch up growth of HC leading to HC comparable to their AGA peers at the age of 6 months. Sex and intrauterine growth are associated with weight, length, and HC at 6 months. Maternal height only shows any influence on the length of their peers at the age of 6 months.

## Supplementary Information

**Additional file 1: Table S1.** Baseline characteristics of infants included in the final analyses vs. those lost to follow up or excluded due to failure of exclusive breastfeeding. **Table S2.** Weight, length and head circumference measurement from birth to 6 months of age.

## Data Availability

The datasets used and/or analysed during the current study are available from the corresponding author on reasonable request.

## Abbreviations

AGA: Appropriate-for-gestational-age
AIDS: Acquired immunodeficiency syndrome
BMI: Body mass index
BRF: Breastfeed
CT: Computed tomography
CUG: Catch up growth
FOF: Formula fed
GH: Growth hormone
HC: Head circumference
HIV: Human immunodeficiency virus
IGF: Insulin-like growth factor
IQ: Intelligence quotient
IUGR: Intrauterine growth restriction
NICU: Neonatal Intensive Care Unit
SD: Standard deviation
SDS: Standard deviation scores
SGA: Small-for-gestational age
