# Supplementary material for: Growth of exclusively breastfed small for gestational age term infants in the first six months of life: a prospective cohort study
Source: BMC Pediatr. 2022 Feb 1;22:73. doi: 10.1186/s12887-021-03080-6 (PMC8805422; doi:10.1186/s12887-021-03080-6)
Supplement: Supplementary file 1 — Additional file 1: Table S1. Baseline characteristics of infants included in the final analyses vs. those lost to follow up or excluded due to failure of exclusive breastfeeding. Table S2. Weight, length and head circumference measurement from birth to 6 months of age. [file 12887_2021_3080_MOESM1_ESM.zip › Supplementary_Table_S1.docx]

Supplementary Table S1. Baseline characteristics of infants included in the final analyses vs. those lost to follow up or excluded due to failure of exclusive breastfeeding

| **AGA infants** | Excluded (n=15) | Included (n= 39) | p-value |
| --- | --- | --- | --- |
| Weight at birth (g) | 3217.4 ± 268.9 | 3143.4 ± 339.8 | 0.305 |
| Length at birth (cm) | 49.7 ± 1.5 | 49.1 ± 1.9 | 0.162 |
| HC at birth (cm) | 34.5 ± 0.9 | 33.96 ± 1.4 | 0.060 |
| Fenton SDS of weight | -0.25 ± 0.71 | -0.2 ± 0.6 | 0.809 |
| Fenton SDS of length | -0.18 ± 0.82 | -0.2 ± 0.9 | 0.933 |
| Fenton SDS of HC | -0.07 ± 0.80 | -0.1 ± 0.9 | 0.893 |
| C-section, n (%) | 9 (60) | 17 (43.6) | 0.437 |
| **SGA infants** | Excluded (n=20) | Included (n=17) |  |
| Weight at birth (g) | 2363.9 ± 517.4 | 2244.5 ± 387.4 | 0.315 |
| Length at birth (cm) | 46.23 ± 3.1 | 45.4 ± 2.3 | 0.255 |
| HC at birth (cm) | 32.24 ± 1.8 | 31.5 ± 1.5 | 0.083 |
| Fenton SDS of weight | -2.09 ± 0.9 | -2.1 ± 0.7 | 0.977 |
| Fenton SDS of length | -1.57 ± 1.1 | -1.5 ± 1.0 | 0.789 |
| Fenton SDS of HC | -1.34 ± 1.03 | -1.1 ± 1.3 | 0.321 |
| C-section, n (%) | 14 (70) | 8 (47.1) | 0.280 |

AGA: Appropriate for gestational age; SGA: small for gestational for age; SDS: standard deviation score; HC: head circumference; C-section: caesarean section
